# A novel and accelerated method for integrated alignment and variant calling from short and long reads

**DOI:** 10.3389/fbinf.2025.1691056

**Published:** 2026-01-05

**Authors:** Jinnan Hu, Donald Freed, Hanying Feng, Hong Chen, Zhipan Li, Haodong Chen

**Affiliations:** Sentieon Inc., San Jose, CA, United States

**Keywords:** NGS-next generation sequencing, secondary analysis, variant calling, hybrid analysis, machine learning, accelerated analysis

## Abstract

**Background:**

Integrating short- and long-read sequencing technologies has become a promising approach for achieving accurate and comprehensive genomic analysis. Although short-read sequencing (Illumina, etc.) offers high base accuracy and cost efficiency, it struggles with structural variant (SV) detection and complex genomic regions. In contrast, long-read sequencing (PacBio HiFi) excels in resolving large SVs and repetitive sequences but is limited by throughput, higher insertion or deletion (indel) error rates, and sequencing costs. Hybrid approaches may combine these technologies and leverage their complementary strengths and different sources of error to provide higher accuracy, more comprehensive results, and higher throughput by lowering the coverage requirement for the long reads.

**Methods:**

This study benchmarks the DNAscope Hybrid (DS-Hybrid) pipeline, a novel integrated alignment and variant calling framework that combines short- and long-read data sequenced from the same sample. The DNAscope Hybrid pipeline is a bioinformatics pipeline that runs on generic x86 CPUs. We evaluate its performance across multiple human genome reference datasets (HG002–HG004) using the draft Q100 and Genome in a Bottle v4.2.1 benchmarks. The pipeline’s ability to detect small variants [single-nucleotide polymorphisms (SNPs)/indels)], SVs, and copy-number variations (CNVs) is assessed using data from the Illumina and PacBio sequencing systems at varying read depths (5×–30×). Benchmark results are compared to those of DeepVariant.

**Results:**

The DNAscope Hybrid pipeline significantly improves SNP and indel calling accuracy, particularly in complex genomic regions. At lower long-read depths (e.g., 5×–10×), the hybrid approach outperforms stand-alone short- or long-read pipelines at full sequencing depths (30×–35×), reducing variant calling errors by at least 50%. Additionally, the DNAscope Hybrid outperforms leading open-source tools for SV and CNV detection and enhances variant discovery in challenging genomic regions. The pipeline also demonstrates clinical utility by identifying variants in disease-associated genes. Moreover, DNAscope Hybrid is highly efficient, achieving less than 90 min runtimes at single standard instance.

**Conclusion:**

The DNAscope Hybrid pipeline is a computationally efficient, highly accurate variant calling framework that leverages the advantages of both short- and long-read sequencing. By improving variant detection in challenging genomic regions and offering a robust solution for clinical and large-scale genomic applications, it holds significant promise for genetic disease diagnostics, population-scale studies, and personalized medicine.

## Introduction

Over the past decade, next-generation sequencing (NGS) and third-generation sequencing (TGS) have become a cornerstone in genomics research and medical applications, driving significant discoveries in disease mechanisms, population diversity, and personalized medicine strategies (Goodwin et al., 2016; Satam et al., 2023). These advancements were facilitated by improvements in sequencing technologies, including reduced costs, enhanced read lengths, higher base quality, and increased accessibility to laboratories at various sizes.

Highly accurate methods for detecting single-nucleotide polymorphisms (SNPs) and <50 bp insertions or deletions (indels) have been central to genetic disease and tumor diagnostics. Additionally, the adoption of long-read sequencing has enabled better integration of structural variants (SVs; ≥50 bp insertions, deletions, or other rearrangement) into analyses (De Coster et al., 2021; Mahmoud et al., 2019). Although SVs are less abundant than small variants in the human genome, they collectively impact more base pairs and play crucial roles in human evolution and disease (Sudmant et al., 2015). Copy-number variations (CNVs), arising from DNA segment deletions or duplications, represent another form of genomic variation linked to various diseases (Zarrei et al., 2015). Despite these advancements, detecting and interpreting these variants together in an integrated analysis pipeline remain challenging.

Although short-read sequencing technologies (e.g., Illumina, Element Biosciences, MGI, etc.) effectively capture small variations across most of the human genome, they face challenges in difficult-to-map regions and in the detection of structural variant. Studies have demonstrated the limitations of short reads for identifying larger insertions, deletions, and other complex genomic rearrangements (Zook et al., 2020). Long-read sequencing technologies, such as PacBio HiFi, have been proposed to address these limitations. These platforms enable improved detection of complex SVs due to their ability to produce reads exceeding 15 kb in length with current base accuracies ranging from 99% to 99.9% (De Coster et al., 2021; Hoffmann et al., 2024; Amarasinghe et al., 2020). Nevertheless, these technologies are not without challenges. Errors in long-read sequencing often manifest as context-specific insertions and deletions (e.g., homopolymers), complicating the detection of indel variants even with high read coverage (Wenger et al., 2019). Additionally, the high cost of generating long reads, combined with their computational demands, poses barriers to large-scale applications, including population-wide studies and analysis of legacy samples. Many interesting samples slated for long-read analysis already have full-coverage short-read data. By using full-coverage short-read data with long-read data, this new pipeline leverages the strengths of both technologies and allows users to decrease long-read coverage by 2×–3× while simultaneously increasing the accuracy and comprehensiveness of results for each sample.

The complementary error profiles of short- and long-read sequencing technologies have motivated the development of hybrid analysis pipelines that leverage both data types. Initially, such approaches were implemented for *de novo* genome assembly, in which short reads were used to correct errors in long-read assemblies (Zhang et al., 2020; Brown et al., 2021). Several hybrid re-sequencing pipelines have also emerged, including “HELLO,” which utilizes deep learning to perform variant calling using combined alignments of short and long reads (Ramachandran et al., 2021). Another notable pipeline, “blend-seq,” focuses on combining ultralow-coverage long reads (approximately 4× coverage) with standard 30× short reads for cost-effective variant discovery (Magner et al., 2024). Clinically, Variantyx has integrated short- and long-read analyses into a single diagnostic workflow, generating a comprehensive clinical report. This pipeline, however, uses long reads primarily for orthogonal confirmation of variants detected by short reads, leaving opportunities for further integration and optimization (Kaplun et al., 2023).

These existing pipelines independently align short and long reads to reference genomes without exploiting the potential of realignment to add value for variant calling. Moreover, limited attention is given to computational efficiency and speed, making them less viable for clinical settings such as neonatal intensive care units (NICUs) or large-scale cohort analyses.

The Genome in a Bottle (GIAB) Consortium has progressively improved its reference sample variant benchmark. The v4.2.1 variant call set, released in 2022, incorporated linked-reads and long-read sequencing data, expanding high-confidence regions in the GRCh38 assembly from 85% to 92% of the genome. This update introduced difficult-to-map regions and other challenging genomic loci not previously included in the v3.3.2 call set (Wagner et al., 2022a). In addition to the genome-wide SNP/indel benchmark, the GIAB released an SV benchmark (v0.6) (Zook et al., 2020) and a benchmark for challenging medically relevant genes (CMRGs) (Wagner et al., 2022b). In a separate effort, the Telomere-to-Telomere (T2T) consortium has published high-quality assemblies of the HG002 sample (Rhie et al., 2023). The initial assembly leveraged PacBio HiFi and ONT (Oxford Nanopore Technologies) data from the Human Pangenome Reference Consortium (HPRC) and GIAB. Following extensive polishing and validation, the v1.1 diploid assembly achieved near-perfect haplotype phasing and an error rate below one per 10 billion bases (a Phred quality score of Q100) (Hansen et al., 2025). Through alignment of the Q100 assembly to GRCh38, the GIAB team has generated a draft assembly-based benchmark for HG002. This new benchmark provides significantly more small variants and nearly three times the number of confident SV events compared to the earlier GIAB v0.6 SV benchmark (29,167 vs. 9,646) (Saunders et al., 2025). These advancements underscore the importance of choosing technologies and datasets aligned with cutting-edge genomic knowledge for clinical and research applications.

Sentieon has won a variety of awards in the PrecisionFDA Challenges including an award in Truth Challenge V2 for multi-platform analysis (Olson et al., 2022), in which short and long reads were used to improve accuracy. The DNAscope Hybrid (DS-Hybrid) pipeline presented here is a substantial improvement from the PrecisionFDA winning pipeline. Different from the previously published DNAscope pipeline for short reads (Freed et al., 2022a) and the DNAscope LongRead pipeline for long reads (Freed et al., 2022b) or any existing pipelines, this hybrid analysis tool integrates short- and long-read sequencing data from the same sample by realigning short reads using the sample-specific long-read information to deliver comprehensive and accurate variant calling.

**FIGURE 1:**
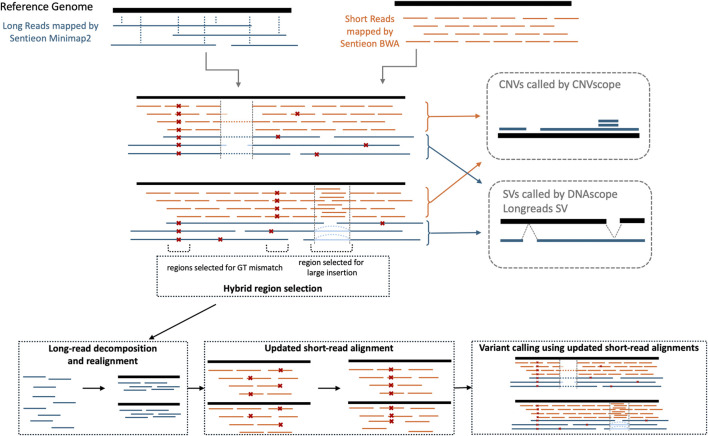
Overview of the processing steps of the DNAscope Hybrid variant calling pipeline.

In this work, we present the DNAscope Hybrid pipeline, which utilizes short- and long-read data from a single sample to achieve highly accurate variant calling. As shown in Figure 1, the DNAscope Hybrid accepts FASTQ or BAM files as input and generates VCF outputs containing SNP, indel, SV, and CNV data. By combining the strengths of both sequencing platforms, the pipeline achieves superior variant detection compared to using either short- or long-read technology in isolation. The DNAscope Hybrid can be used with whole-genome sequencing (WGS) long-read data or with targeted sequencing approaches such as the Twist Alliance Dark Genes Panel (Deserranno et al., 2025). The pipeline’s performance and versatility make it a promising tool for clinical diagnostics, particularly in settings requiring highly accurate, comprehensive results.

Compared with the existing hybrid analysis methods (Ramachandran et al., 2021; Magner et al., 2024; Kaplun et al., 2023) mentioned above, DNAscope Hybrid introduces a novel long- and short-read realignment step designed to enhance performance in complex genomic regions. This approach leverages the read-length advantage of long reads together with the higher depth and indel calling accuracy of short reads, thereby improving overall variant calling accuracy and expanding confident variant calls into more challenging genomic areas. Existing pipelines do not perform a comprehensive realignment and therefore fail to fully realize the highest accuracy from short- and long-read data.

To evaluate the performance of the DNAscope Hybrid pipeline, we benchmark the pipeline output using a variety of benchmarks. We benchmark the small-variant (SNV and indel) VCF using the GIAB v4.2.1, CMRG, and Q100 benchmarks. SVs identified by the pipeline are assessed using the CMRG and draft Q100 SV benchmarks. CNVs are assessed using a benchmark constructed from the Q100 SV benchmark. The runtime of the DNAscope Hybrid pipeline is assessed by running the pipeline using a public cloud server.

## Results

### Small variants (SNPs and indels)

To evaluate the accuracy of the DS-Hybrid pipeline at varying depths, we used the HG002 sample and a PacBio HiFi (PB) dataset down-sampled to depths of 5×, 7.5×, 10×, 15×, 20×, and 30×, paired with 35× Illumina (ILMN) short-read data. To assess the accuracy contribution of short reads, we also analyzed each depth of PB datasets independently without short reads using the DNAscope LongRead (DS-LR) pipeline. Additionally, we included other datasets for comparison: Illumina (35×) data analyzed using DRAGEN v4.2 and PacBio HiFi (30×) analyzed using DeepVariant (DV) v1.8.0.

**FIGURE 2:**
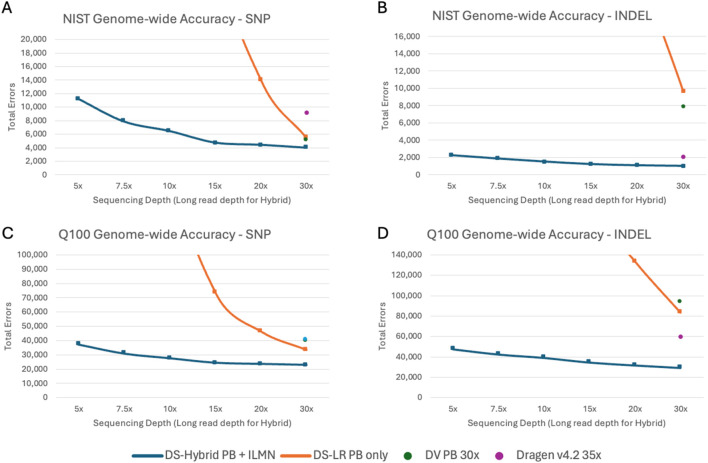
Genome-wide accuracy—total errors of **(A)** SNP in GIAB v4.2.1; **(B)** indel in GIAB v4.2.1; **(C)** SNP in draft Q100; **(D)** indel in draft Q100. DS-Hybrid PB + ILMN and DS-LR PB only are shown with curves covering 5×–30× long-read depths. DV PB and DRAGEN are shown at full depth.

We initially investigated genome-wide accuracy using the NIST v4.2.1 benchmark (Figures 2A,B; Supplementary Table S1). This analysis demonstrated that higher depths of long-read sequencing yield greater accuracy, with the highest accuracy observed in the combined 30× PB + 35× ILMN datasets. Furthermore, hybrid indel accuracy is higher than that of the other evaluated methods, even when using only 7.5× coverage for long reads. Notably, the DNAscope Hybrid pipeline improves SNP and indel accuracy compared to any single-technology pipeline.

The current cost for DNA extraction and library preparation is approximately $735 USD for PacBio HiFi and $135 USD for Illumina [the service cost is from a single service provider (UC San Diego Genomics Core, 2025) as it will differ elsewhere]. Sequencing costs are approximately $330 for 10× PacBio HiFi coverage (Pacific Biosciences, 2024) and $200 for 30× Illumina coverage (Illumina, 2023). Therefore, generating a combined dataset of 10× PacBio HiFi + 30× Illumina would result in a total wet laboratory cost comparable to generating 20× PacBio HiFi data alone.

Based on these data, 10× of PacBio and 35× of Illumina have a good balance between the cost of reagents and results. At this coverage level, the pipeline has 1,527 indel errors and 6,467 SNP errors when evaluated on the GIAB v4.2.1 benchmark, for F1 values of 0.9985 and 0.9990, respectively.

Comparing the draft Q100 and the v4.2.1 benchmarks, total errors are much higher with the draft Q100 benchmark as it contains more challenging regions (Figures 2C,D; Supplementary Table S2), making it more suitable for benchmarking new high accuracy variant callers. In the draft Q100 benchmark, the hybrid pipeline has fewer errors than single-technology pipelines for both SNPs and indels. Comparing the hybrid pipeline with 10× long-read coverage with the draft Q100, SNP errors are reduced by 30% relative to the next best pipeline (DS-LR) and indel errors are reduced by 35% relative to the next best pipeline (DRAGEN).

**FIGURE 3:**
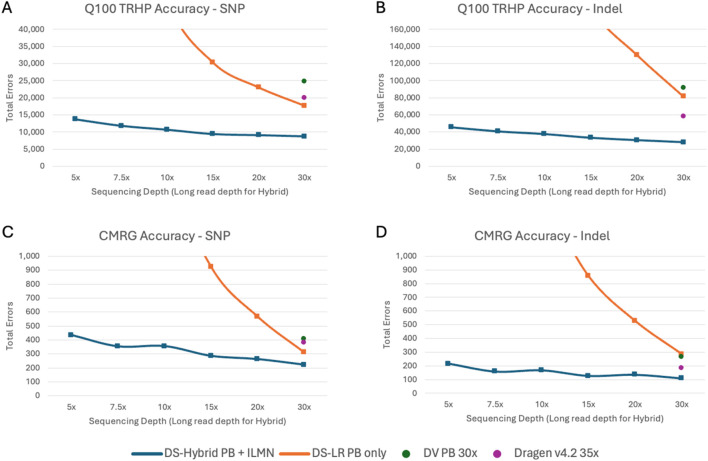
Stratified region accuracy—total errors of **(A)** SNP and **(B)** indel in tandem repeat and homopolymer (TRHP) regions in the Q100 benchmark. **(C)** SNP and **(D)** indel in the challenging medically relevant genes (CMRG) benchmark. DS-Hybrid PB + ILMN and DS-LR PB only are shown with curves covering 5×–30× long-read depths. DV PB and DRAGEN are shown at full depth.

To better understand the variant calling accuracy improvement in the hybrid pipeline, we performed a stratified analysis across GA4GH stratification regions (Krusche et al., 2019). Variant calling accuracy, as measured with the draft Q100 benchmark at annotated tandem repeat and homopolymer (TRHP) regions, is shown in Figures 3A,B. We additionally assessed variant calling accuracy using the CMRG benchmark for HG002 (Figures 3C,D). The DNAscope Hybrid pipeline has improved accuracy at TRHP regions, and the DNAscope Hybrid pipeline with 5× long-read coverage outperforms the other benchmarked pipelines. Short reads frequently fail to map to tandem repeats correctly, and long reads have less accurate resolution of homopolymers. By using the two data types in a complimentary way, the hybrid method helps resolve both sources of error. CMRG regions, which encompass 273 medically relevant genes, demonstrated substantial benefits from hybrid short- and long-read data. Long reads alone cannot capture each variant correctly, whereas the hybrid pipeline still showed its improved accuracy, especially for indels. The improved accuracy will likely lead to an improved diagnostic rate and other clinical utility.

**FIGURE 4:**
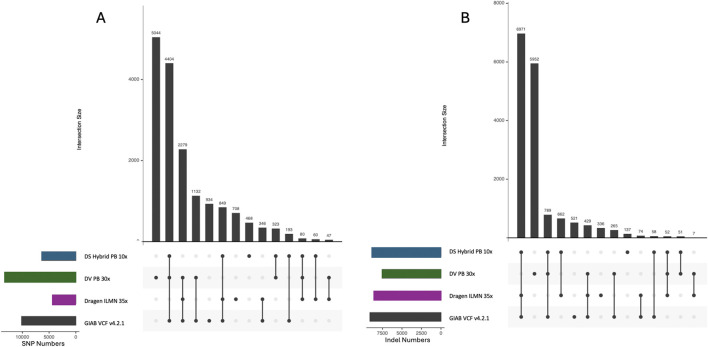
UpSet plots of **(A)** SNPs and **(B)** indels, including three benchmarked pipelines and the GIAB v4.2.1 benchmark VCF. The intersection (bar) of variants identified by all three pipelines that are also in the GIAB VCF is removed, and the remaining intersection categories are sorted by size. Call set sizes for each pipeline are displayed on the lower left panels. The UpSet plots have slightly different results from those in Figure 2 and Supplementary Table S1, due to the different comparison approaches that were used.

To better understand the differences between the evaluated pipelines, we compared the intersection of the DNAscope Hybrid VCF, VCFs from the stand-alone short- or long-read pipelines, and the GIAB v4.2.1 benchmark VCF (Figure 4). Variants detected by all pipelines that are also present in the benchmark VCF represent the highest proportion but are not displayed. ILMN detected fewer SNPs, with a higher number of false negatives, whereas PB had a higher rate of false-positive SNPs. Variants missed by short-read pipelines were mainly attributed to low mappability and poor coverage, whereas those missed by long-read pipelines were primarily due to inherent limitations in base calling accuracy, particularly at homopolymer indels.

**FIGURE 5:**
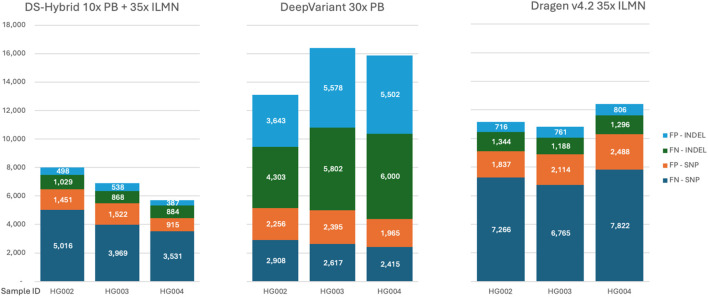
SNP/indel accuracy over HG002–HG004 reference samples. False-positive and false-negative counts for SNPs and indels are listed separately.

Although the DNAscope Hybrid pipeline has excellent performance on HG002, we wanted to further assess the performance on additional datasets to ensure that the approach used by the pipeline extends to other samples. We then applied the DNAscope Hybrid pipeline to two additional GIAB samples. The results, measured as SNP and indel combined total errors (FP + FN), were compared to those of the platform-recommended pipelines for Illumina short reads and PacBio long reads (Figure 5). This figure shows that DNAscope Hybrid call sets are consistently more accurate than short- or long-read-only call sets in the tested sample, highlighting its robustness and adaptability.

### Structural variants

To evaluate structural variant (SV) accuracy, we analyzed down-sampled long- and short-read datasets and evaluated variant calling accuracy using the draft Q100 or CMRG SV benchmarks. For the hybrid SV pipeline, only long-read information was utilized. Other benchmarked pipelines include PacBio SV calls generated using Pbsv and 35× ILMN SV calls generated using DRAGEN v4.2.

**FIGURE 6:**
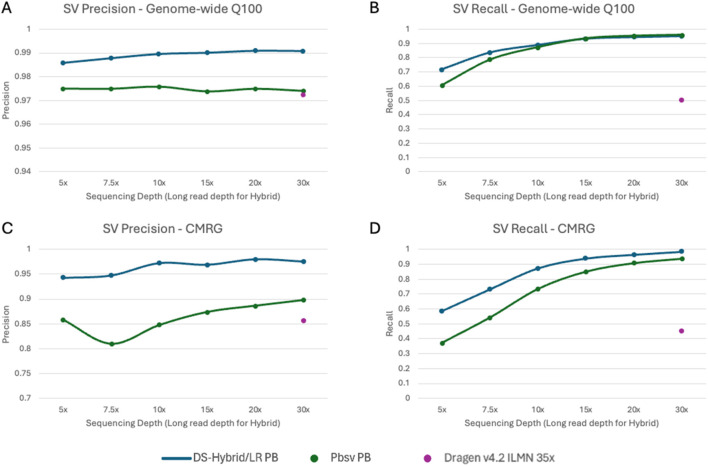
Structural variant (SV) accuracy as measured by **(A)** precision on the draft Q100 benchmark; **(B)** recall on the draft Q100 benchmark; **(C)** precision on the CMRG SV benchmark; **(D)** recall on the CMRG SV benchmark. PB-Hybrid/LongRead and Pbsv PB are shown as curves covering 5×–30× long-read depths. DRAGEN ILMN is shown at full-depth accuracy.

The genome-wide draft Q100 SV benchmark complements the information in the SNP/indel accuracy curves (Figures 6A,B; Supplementary Table S6). Although short-read pipelines demonstrate high accuracy for SNP and indel detection, these pipelines have lower accuracy for SV calling, particularly for SV recall. In contrast, long-read pipelines have higher measured SV accuracy, even at lower depths, and achieved saturation in performance at a depth approximately 15×–20×. The DNAscope Hybrid/LR pipeline outperformed Pbsv in this benchmark. To further validate pipeline performance, we assessed SV accuracy using the CMRG SV benchmark (Figures 6C,D; Supplementary Table S7). Performance on the CMRG SV benchmark was consistent with the performance observed in the larger draft Q100 SV benchmark, underscoring the advantage of long-read sequencing in SV detection.

**FIGURE 7:**
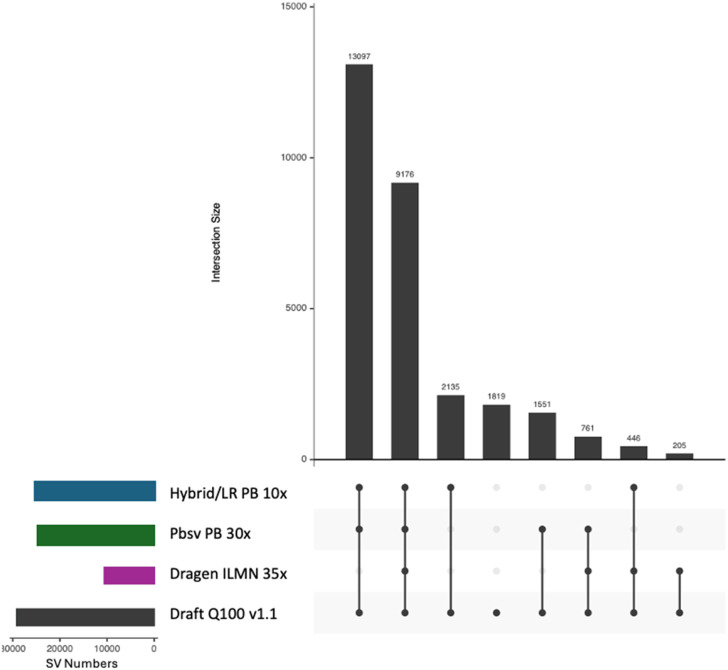
UpSet plot of SVs for the three benchmarked pipelines and the draft Q100 benchmark VCF. Intersection categories are sorted by size. Call set sizes for each pipeline are displayed in the lower left panel.

We further analyzed the intersection of the SV call sets with the draft Q100 SV benchmark (Figure 7). This analysis highlights the substantial number of structural variants missed by the short-read pipeline. These omissions primarily stem from the inherent limitations of short reads, particularly their inability to span longer SVs.

### Copy-number variation

**FIGURE 8:**
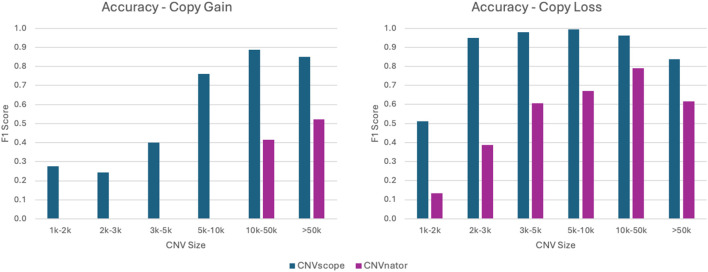
CNV accuracy benchmark. CNVscope serves as the CNV caller in the DNAscope Hybrid and short-read pipelines. CNVnator results are shown for comparison.

CNV refers to genetic differences between individuals involving the loss or gain of specific DNA regions. The current version of the DNAscope Hybrid pipeline utilizes the recently released Sentieon CNVscope tool, which relies solely on short-read data for CNV detection. To evaluate its performance, we benchmarked CNVscope accuracy using the HG002 Q100 benchmark (Figure 8; see methods). We also benchmarked CNVnator accuracy for comparison.

The Sentieon pipeline consistently demonstrated higher accuracy across nearly all event sizes, including the technically challenging <10k events, where CNVnator and most other tools struggle to achieve high accuracy. This suggests that DNAscope Hybrid offers significant improvements in CNV detection.

### Overall variant counts in HG002

**FIGURE 9:**
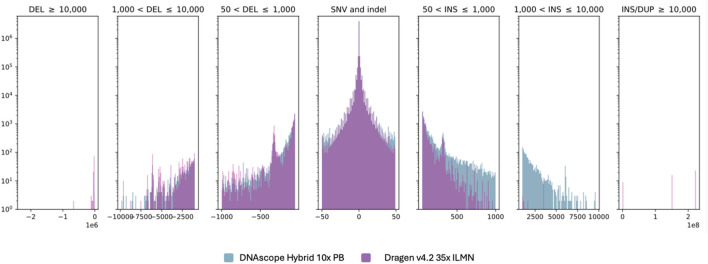
Size distribution of small and structural variants identified by the DNAscope Hybrid pipeline on 10× PB + 35× ILMN and by DRAGEN on 35× ILMN.

DNAscope Hybrid is a comprehensive variant calling pipeline capable of detecting SNVs, short indels (<50 bp), and longer indels. When compared with the short-read DRAGEN pipeline, the hybrid pipeline identified a notably higher number of variants, particularly large insertion events (Figure 9). This increase is primarily attributable to the additional information provided by long-read data integrated into the hybrid workflow.

### Validation using selected variants in clinically relevant genes and simulated pathogenic variants

**FIGURE 10:**
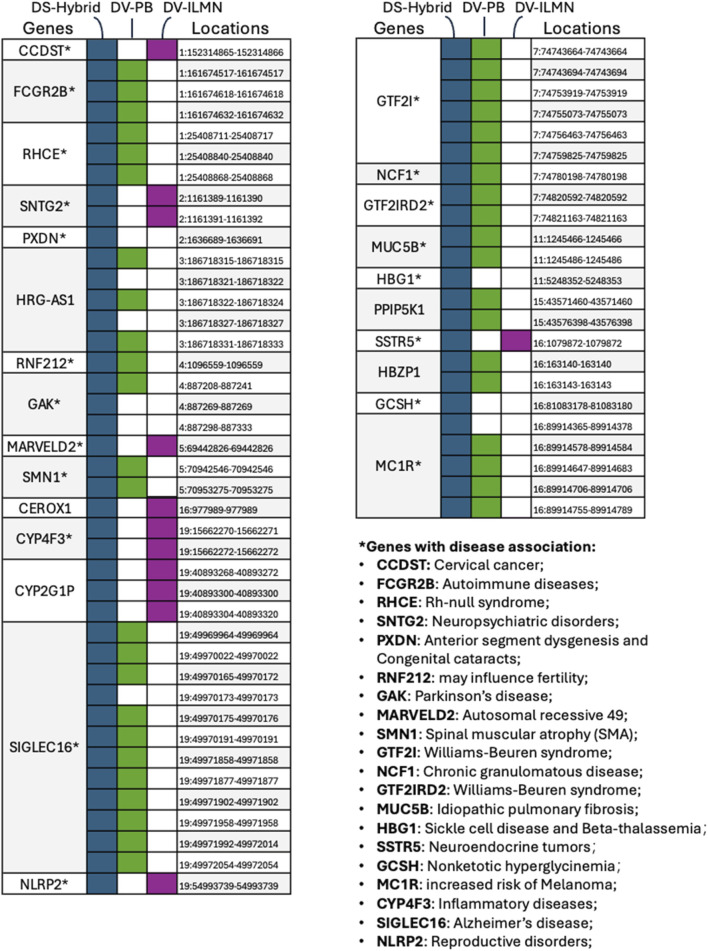
Identification landscape of selected SNPs/indels missed by different pipelines. Variants were selected based on the intersection of HG002 CMRG exonic variants and those identified by one or two of the three pipelines. Many of the CMRG genes have well-documented disease associations, as shown in the right panel.

To evaluate the clinical utility of the DNAscope Hybrid pipeline, we further analyzed HG002 variants in the CMRG benchmark. In particular, HG002 CMRG variants were annotated using VEP (McLaren et al., 2016), and those associated with exons were selected for comparison across three pipelines: 1) the DNAscope Hybrid with 10× PB and 35× ILMN; 2) DeepVariant 30× PB; and 3) DeepVariant 35× ILMN. Variants detected using only one or two of these pipelines are shown in Figure 10. The DNAscope Hybrid pipeline identified all 64 variants, whereas DV PB and DV ILMN failed to capture some, with nine variants exclusively detected by the hybrid pipeline.

We also analyzed a previously published set of clinically relevant germline variants identified from 100 real patient samples (Höps et al., 2025). Some variants in these patients were initially identified by whole-exome sequencing, whereas other variants were identified using other molecular diagnostic approaches, including Sanger sequencing and molecular ligation-based probe amplification (MLPA), as these variants are difficult to detect from traditional short-read sequencing. From this dataset, we selected all 42 SNP/indel variants for further analysis. Of these, 36 were classified as “difficult to detect by short-read sequencing” due to their location within homologous regions or pseudogenes. The remaining six variants were included because of complexities such as structural rearrangements, homopolymer repeats, imprinting effects, phasing challenges, or location within pseudoautosomal regions. Notably, even long-read sequencing alone failed to identify three of these variants without manual inspection in IGV. Additional details for all 42 variants are provided in the supplementary table of a previously published study (Höps et al., 2025).

We generated simulated Illumina short reads at 30× coverage and PacBio HiFi long reads at 10× coverage and processed the simulated data through the DNAscope Hybrid pipeline. The DNAscope Hybrid pipeline successfully detected all 42 SNPs/indels. Among the identified variants, we focused on those previously reported in clinical cases, as shown in Table 1.

**TABLE 1:** Selected NGS challenging clinical SNPs/indels carried by patients diagnosed using traditional detection methods.

Patient sample ID	Case reported associated with this variant	Variant details	Gene locus	Original diagnostic method	Detected using DNAscope Hybrid
P3-E11	Hearing loss (RCV000151940.7)	Chr15(GRCh38):g.43600609_43600610delinsAG	STRC	Exome sequencing	True
P10-A4	Congenital adrenal hyperplasia (CAH)	Chr6(GRCh38):g.32040535C>T	CYP21A2	Sanger sequencing	True
P10-B4	Congenital adrenal hyperplasia (CAH)	Chr6(GRCh38):g.32039081C>G	CYP21A2	Sanger sequencing	True
P11-F11	Nonsyndromic high myopia and reduced cone function	OPN1LW: LVAVA combination	OPN1LW; OPN1MW	Amplicon-based long-read sequencing	True
P50-D1	Cone dysfunction syndromes	1 copy OPN1LW: MIAVA combination	OPN1LW; OPN1MW	Molecular ligation-based probe amplification (MLPA) + Sanger sequencing	True
P50-A5	Hereditary breast and ovarian cancer syndrome (HBOC)	Chr17(GRCh38):g.43093577del	BRCA1	Single-molecular molecular inversion probes (smMIPs)	True
P50-B5	Hereditary breast and ovarian cancer syndrome (HBOC)	Chr13(GRCh38):g.32333291dup	BRCA2	Single-molecular molecular inversion probes (smMIPs)	True

**FIGURE 11:**
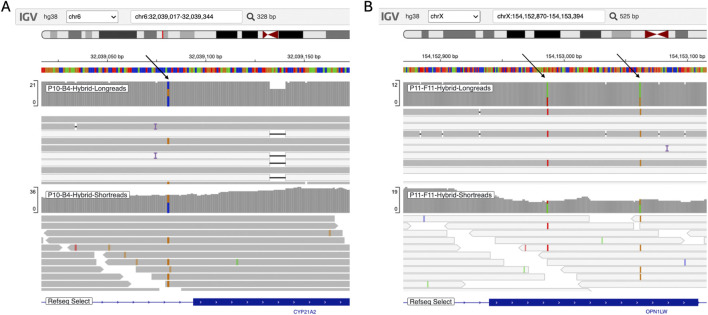
IGV screenshot showing variants: **(A)** Chr6(GRCh38):g.32039081C>G, carried by Patient P10-B4, and **(B)** OPN1LW: LVAVA combination, carried by Patient P11-F11. The upper tracks display the mapped results of simulated long reads, and the lower tracks show realigned short reads. Target variants are indicated by black arrows.

A notable example is Patient P10-B4 (Figure 11A), who was diagnosed with congenital adrenal hyperplasia (CAH) caused by an SNP in intron 2 of the *CYP21A2* gene. This variant disrupts normal splicing, leading to impaired 21-hydroxylase enzyme function. The *CYP21A2* gene has a highly similar pseudogene (*CYP21A1P*), which creates challenges for short-read sequencing alignment due to their high sequence homology. Traditionally, Sanger sequencing was the only reliable method for detecting such variants. However, in this case, long-read assembled haplotype data were used to guide alignment, enabling accurate mapping of short reads and providing strong support for variant identification.

Figure 11B shows simulated read data from patient P11-F11, who carries the LVAVA combination, which affects five key amino acid positions in exon 3 of the *OPN1LW* gene. OPN1LW plays a crucial role in the spectral tuning of the red-sensitive photopigment, and mutations are associated with color vision impairment. The two critical DNA substitutions defining the LVAVA variant are highlighted by arrows, indicating their significance in modifying the opsin protein’s function. *OPN1LW* has a high degree of sequence identity to the other opsin genes, *OPN1MW* and *OPN1SW*, which creates challenges for short-read alignment across the opsin genes. The hybrid pipeline overcomes the difficulty in short-read alignment, providing sufficient coverage for accurate variant identification.

### Compute resource benchmark

A major challenge in whole-genome sequencing secondary analysis is the long runtime, high cost of compute, and requirements for specialized hardware for obtaining an adequate TAT. Sentieon software addresses these issues by running efficiently on commodity (x86 or ARM) CPU servers or workstations, offering accelerated runtimes, improved consistency, and high accuracy compared to other tools.

To assess the runtime of Sentieon software, we tested three Sentieon pipelines—the DNAscope Hybrid pipeline with 10× PacBio HiFi and 35× Illumina data, DNAscope LongRead (PB) with 30× PacBio HiFi data, and DNAscope with 30× Illumina data. The benchmark assessed the runtime performance of alignment, preprocessing, and SNP/indel/SV/CNV calling. A 120-thread Azure instance (Standard HB120rs v3) was used as the computation environment. The results for runtime, core-hours, and compute cost are shown in Table 2. The DNAscope LongRead and DNAscope pipeline runtimes were previously published (Microsoft Healthcare and Life Sciences Blog, 2024).

**TABLE 2:** Compute resource benchmark for DNAscope pipelines. The benchmark environment is Azure Standard HB120rs v3 (120 vCPUs, 456 GiB memory, 512 GB premium SSD); runtime and on-demand compute cost are displayed. The DNAscope Hybrid pipeline outputs SNP/indel/SV/CNV, the DNAscope LongRead pipeline outputs SNP/indel/SV, and DNAscope short reads output SNP/indel/CNV.

Pipeline	DNAscope Hybrid	DNAscope LongRead	DNAscope (short reads)
Dataset	10× PB + 35× ILMN	30× PB	30× ILMN
Alignment (min)	18.8	11.5	9.7
Preprocessing (min)	2	0	1.4
Variant calling (min)	65	29.8	7.8
Total runtime (min)	85.8	41.2	18.9
Core-hours	171.7	82.5	37.8
On-demand ($)	5.2	2.5	1.1
Spot ($)	0.52	0.25	0.11

The DNAscope Hybrid pipeline is actively being developed, with future releases expected to show incremental improvements in computational efficiency and accuracy. Benchmarking results indicate that all three Sentieon pipelines completed the FASTQ-to-VCF analysis in approximately 20 min to less than 90 min for a cost of between $0.11 and $5.20 depending on data and spot or on-demand pricing.

## Methods

### Datasets used in this study

FASTQ files were downloaded from publicly available datasets:

PacBio: Human whole-genome sequencing datasets from the Revio system for the Genome in a Bottle trio HG002 + HG003 + HG004, with one Revio SMRT Cell per sample replicate (PacBio, 2022).

Illumina: pFDA Truth Challenge V2 (PrecisionFDA, 2025).

Benchmark VCFs: SNP/indel: NIST V4.2.1; draft Q100 V0.019; CMRG V1.00 and SV: Q100 V0.019; CMRG V1.00. An overview of the tools benchmarked in this study is provided in Table 3.

**TABLE 3:** Sentieon tools and alternative tools in each benchmarking category.

Benchmarking category	Sentieon pipeline or module	Alternative tools for comparison
Small variants (SNPs and indels)	DNAscope Hybrid (DS-Hybrid) DNAscope LongRead (DS-LR)	DeepVariant for PacBio (DV-PB) DRAGEN
Structural variants (SVs)	DNAscope Hybrid (DS-Hybrid) DNAscope LongRead (DS-LR)	pbsv DRAGEN
Copy-number variation (CNV)	CNVscope	CNVnator
Overall variant counts	DNAscope Hybrid (DS-Hybrid)	DRAGEN
Variants in clinically relevant genes	DNAscope Hybrid (DS-Hybrid)	DeepVariant for PacBio (DV-PB) DeepVariant for Illumina (DV-ILMN)

### Pipelines and tools benchmarked in this study

### DNAscope Hybrid pipeline overview

The Sentieon DNAscope Hybrid pipeline is designed to process and integrate both short and long sequencing reads from the same sample, achieving the most comprehensive and accurate variant calling results. This integrated approach ensures that variant calling accuracy surpasses the results obtained by processing short or long reads separately. The pipeline takes FASTQ or BAM files as input and produces SNP, indel, SV, and CNV calls in VCF format as output. It can be applied to any human whole-genome sequencing assay and has potential for extension to other applications, such as whole-exome sequencing, CMRG sequencing, or HLA analysis.

As previously described (Freed et al., 2022a), Sentieon DNAscope is a germline variant caller that performs haplotype-aware germline variant calling using an approach similar to the GATK HaplotypeCaller. In brief, the software identifies active regions or regions of the genome that are likely to contain germline genetic variation. Reads are trimmed to the active region, and read haplotypes are generated using a local assembly. Reads are then aligned to the generated haplotypes using a statistical model, generating a matrix of read likelihoods for each haplotype, which are then marginalized over the variant alleles to generate read likelihoods for each allele. The alleles are then output as a VCF of candidate variants, and the candidate variants are genotyped using a machine-learned model that incorporates variant annotations as model features.

As shown in Figure 1, the DNAscope Hybrid pipeline aligns short reads to the reference genome using Sentieon BWA and aligns long reads using Sentieon Minimap2. In the Sentieon DNAscope Hybrid pipeline, we have extended DNAscope’s variant calling approach using a multi-stage data processing pipeline. The pipeline performs an initial pass of variant calling using the combined short- and long-read data with DNAscope using sensitive variant calling parameters. After the initial pass of variant calling, specific regions are selected for additional investigation. One source of these regions is the “hybrid_select.py” script, which selects sites for further investigation if the long-read data have adequate coverage (at least two reads by default) and if there is a genotype discrepancy between the short- and long-read data using DNAscope’s Bayesian statistical model. Regions containing short-read alignments with a MapQ of 0, unmapped reads, and large insertions in the long-read alignments are also selected for additional investigation.

Once regions are selected for additional investigation, the pipeline uses a sophisticated procedure to correctly place short-read alignments. Long-read alignments across the selected regions are split into smaller sequences and aligned back to the reference genome using Sentieon BWA. The split long-read alignments are then analyzed to determine the optimal placement of short reads, moving the short-read alignments to their most likely location given the long-read information. This procedure optimally utilizes the long reads and the human reference genome to place the short reads correctly. After this realignment procedure, a second pass of variant calling is performed across regions with updated short-read alignments using the realigned short reads.

VCF files from the first and second passes of variant calling are merged, annotated and genotyped, and filtered using a DNAscope machine learning model, similar to the Sentieon DNAscope pipeline (Freed et al., 2022a). The machine learning model used in the DNAscope Hybrid pipeline is trained with a specific combination of short- and long-read sequence data (Illumina and PacBio HiFi, for example), using the GIAB v4.2.1 benchmark with chromosome 20 and the HG003 sample held out from model training. After genotyping and filtering, variants are normalized using “bcftools norm” to generate the final output VCF containing single-nucleotide variants and small indels (generally less than 50 bp).

Structural variants (indels larger than 50 bp) are detected using the DNAscope LongReadSV algorithm, and CNVs are called using short-read-only CNVscope algorithm. A detailed description of these two algorithms is provided below.

The DNAscope Hybrid pipeline is implemented in the Sentieon software package, a highly optimized, commercial suite of tools for biological data processing. The pipeline uses multiple tools within the Sentieon software package, and a command line interface (sentieon-cli) has been developed to allow users to easily run the full hybrid pipeline by specifying the input, output, and key parameters (Sentieon, 2025a). The sentieon-cli calls the sentieon software package and the open-source tools bcftools, bedtools, and samtools from the user’s PATH when running the DNAscope Hybrid pipeline.

 sentieon-cli dnascope-hybrid [-h] \
 -r REFERENCE \
 --sr-aln SR_ALN [SR_ALN …] \
 --lr_aln LR_ALN [LR_ALN …] \
 -m MODEL_BUNDLE \
 [-d DBSNP] \
 [-b DIPLOID_BED] \
 [-t NUMBER_THREADS] \
 sample.vcf.gz

In addition to the DNAscope Hybrid pipeline, the following Sentieon pipelines and modules were utilized in this benchmark: BWA/MM2 alignment (which supports machine learning models), DNAscope LongRead, DNAscope LongRead SV, CNVscope, and hap-eval.

### Sentieon alignment

Sentieon released an accelerated version of BWA-MEM (Li and Durbin, 2009; Li, 2013) in 2017 (Freed et al., 2017) and followed with an accelerated version of Minimap2 (Li, 2018) in the 202010.04 release. These tools provide results consistent with the open-source versions but deliver 2×–3× faster performance. In the 202308 release, a new version of these two alignment tools was introduced, improving whole-genome sequencing alignment runtimes by approximately 2× using a model file to optimize compute resources.

For this benchmark, Illumina NovaSeq 6000 reads from HG002 samples and other two GIAB samples (downloaded from the PrecisionFDA Truth V2 challenge) were mapped to the GRCh38 reference genome (GCA_000001405.15_GRCh38_no_alt_analysis_set_maskedGRC_exclusions_v2.fasta). Reads from PacBio Revio were mapped to the GRCh38 reference genome using Sentieon Minimap2.

### Short variant calling

In addition to the DNAscope Hybrid pipeline, down-sampled long-read datasets were also analyzed using the DNAscope LongRead pipeline (Freed et al., 2022b). Originally published in collaboration with PacBio in 2022, DNAscope LongRead performs mapping and phasing and utilizes pre-trained machine learning models to correct sequencer-specific error patterns and accurately call short variants. Subsequent releases added support for ONT reads, further enhancing pipeline performance. The DNAscope LongRead pipeline can also be run via Sentieon-cli, with the following command line:

 sentieon-cli dnascope-longread [-h] \
 -r REFERENCE \
 --fastq INPUT_FASTQ … \
 --readgroups READGROUP … \
 -m MODEL_BUNDLE \
 [-d DBSNP] \
 [-b DIPLOID_BED] \
 [-t NUMBER_THREADS] \
 [-g] \
 --tech HiFi|ONT \
 [--haploid-bed HAPLOID_BED] \
 sample.vcf.gz

DeepVariant (v1.8.0) (Poplin et al., 2018) was used to generate variants from long-read-only data, using its default settings. DRAGEN accuracy metrics were generated from VCF files downloaded from a recently published work (Behera et al., 2024).

Benchmarking of SNV and Indels was conducted using hap.py with the GIAB V4.2.1, draft Q100, and CMRG benchmark VCFs and BED files.

### Structural variant calling and benchmarking with hap-eval

The DNAscope LongRead SV caller is integrated into the DNAscope Hybrid pipeline. It performs haplotype-resolved SV calling and works well with both PacBio HiFi and ONT data, supporting various sequencing chemistry versions and base callers. Pbsv (v2.10.0) (Pacific Biosciences, n. d.) was also used to generate long-read-only SVs. DRAGEN v4.2 SVs were obtained from an earlier publication (Behera et al., 2024).

Benchmark of SVs was performed using “hap-eval” (Sentieon, 2025b), an open-source structural variant benchmarking tool developed by the Sentieon team. Sentieon developed hap-eval to address some limitations of the popular tool “Truvari” (English et al., 2022). Older versions of Truvari performed a pairwise comparison of variants, without accommodation for multiple nearby variants or multi-allelic sites. Hap-eval assembles multiple variants into haplotypes and conducts haplotype-based comparisons. This VCF comparison engine is assembly-based and compares SV haplotypes at a single-base resolution. Hap-eval works in four steps: 1) combine base and comparison calls in sorted order; 2) create comparison chunks; 3) perform a 1-to-1 comparison within each chunk between base and comparison calls to create a match matrix; and 4) make calls based on the matching matrix.

### CNV calling

In DNAscope Hybrid, CNV identification is performed by CNVscope, a short-read WGS CNV caller first released in Sentieon version 202308.03. CNVscope is designed for germline CNV calling across diploid chromosomes, identifying events greater than 1 kb in length from full-coverage short-read data. The CNVscope algorithm uses read-depth profiling, normalization, feature collection, and segmentation to identify CNV events. Identified events are then filtered using a pre-trained machine learning model. The model was trained and tested using datasets developed from the HPRC and T2T assemblies, including the Q100 assembly. CNVscope uses a reference-independent approach and can be used with hg38, b37, or other high-quality reference genome assemblies of diploid organisms.

 sentieon driver \
 -t NUMBER_THREADS \
 -r REFERENCE \
 -i DEDUPED_BAM \
 --algo CNVscope \
 --model ML_MODEL/cnv.model \
 TMP_VARIANT_VCF
 sentieon driver \
 -t NUMBER_THREADS \
 -r REFERENCE \
 --algo CNVModelApply \
 --model ML_MODEL/cnv.model \
 -v TMP_VARIANT_VCF \
 VARIANT_VCF

We also generated CNV calls using CNVnator (v0.4.1) (Abyzov et al., 2011). Benchmarking of CNVs was conducted using an in-house developed script. The evaluation between the call and truth considers a call to be a true positive (TP) if it has at least 30% overlap with a truth interval, and both the truth and call variant had a consistent direction (gain or loss). An expanded truth interval is considered if the CNV event occurs at a segmental duplication region.

A primary challenge in benchmarking CNV callers is the absence of a genome-scale, high-quality CNV truth set. To address this gap, we developed a novel benchmark by leveraging the GIAB Q100 SV truth set for HG002—the most complete diploid human genome benchmark currently available. Our approach is predicated on the principle that many large-scale indels are functionally CNV events, such as tandem repeat expansions or contractions. The systematic procedure for converting these long indels into a robust CNV benchmark is as follows:

1. Identify the operative sequence unit: For each long indel, the alternate sequence is first analyzed to identify its canonical repeating unit. This step is crucial because a significant fraction of functional CNVs arise from changes in large repeating units. If no repetitive pattern is detected, the entire indel sequence is treated as the single operative unit for the downstream analysis.
2. Event classification (gain or loss): The nature of the indel dictates its classification. A long deletion is straightforwardly interpreted as a CNV loss at its genomic locus. For a long insertion, we must determine the origin of the inserted sequence to classify it as a CNV gain. To achieve this, the inserted unit is aligned to the reference genome, first targeting the immediate flanking regions of the insertion site (local alignment). A match here indicates a tandem duplication. If no local match is found, a global alignment against the entire reference genome is performed to locate the source of an interspersed duplication. A successful match in either step confirms a CNV gain. If no match is found, this insertion is not considered a CNV gain event.
3. Copy number calculation: The absolute copy number is determined by quantifying the number of operative units within the indel sequence, integrated with the event’s genotype (e.g., homozygous or heterozygous).
4. Boundary refinement: Finally, the genomic interval of the newly defined CNV event may be expanded. This adjustment is based on the sequence context of the matched reference region, ensuring that the final benchmark interval accurately encompasses the entire repetitive element for robust evaluation. This also provides critical information for accurately assessing CNV calls, which may not exactly coincide with the CNV gain locus but fall within the general repeating environment.

This systematic conversion ensures that our CNV benchmark is biologically relevant and robustly derived from an assembly-based SV benchmark.

## Discussion

Here, we present the DNAscope Hybrid pipeline, a robust, fast, and accurate pipeline for combined short- and long-read data. The DNAscope Hybrid pipeline uses a novel approach, with long-read haplotypes guiding short-read alignment. We demonstrate that this approach enables higher variant calling accuracy than single-technology pipelines. In addition to SNVs/indels, the hybrid pipeline incorporates specialized callers for structural and copy-number variation to enable accurate detection of major types of genetic variation.

Hybrid secondary analysis pipelines have emerged to harness the complementary strengths of short- and long-read sequencing technologies. Recently developed hybrid pipelines include “HELLO (Ramachandran et al., 2021),” which utilizes deep learning for variant calling from combined alignments, and “blend-seq (Magner et al., 2024),” which combines ultralow-coverage long reads with standard-depth short reads for cost-effective variant discovery. Clinically, Variantyx (Kaplun et al., 2023) has implemented a unified workflow that integrates both data types into diagnostic reporting, although long reads in this pipeline mainly serve as orthogonal confirmation of short-read variants. Despite these advances, most existing hybrid pipelines align short and long reads independently, failing to utilize the personalized information contained in the long-read alignments, which limits accuracy improvements. In contrast, the DNAscope Hybrid pipeline introduces a novel realignment step that improves performance in complex genomic regions by leveraging long-read length and short-read depth and indel accuracy. The pipeline achieves enhanced computational efficiency through multiple design optimizations, along with focusing processing on genomic regions that benefit most, and other systematic improvements that collectively make the pipeline more efficient than existing alternatives.

Variant calls from the DNAscope Hybrid pipeline and other tools were benchmarked using multiple samples and multiple benchmark datasets. We utilized three samples (HG002, HG003, and HG004) from the GIAB v4.2.1 benchmark to confirm that our method works well across multiple samples and is not overfit to the samples used in model training (HG003 is held out during the training process). We also tested the performance of these tools using the CMRG benchmark and the draft Q100 benchmark. The CMRG benchmark contains genomic regions that are difficult to resolve with short-read sequencing, whereas the draft Q100 benchmark uses an assembly-based approach to extend into regions that are difficult to resolve with traditional mapping-based approaches. Taken together, these benchmarks validate the performance of the DNAscope Hybrid pipeline as a robust pipeline for the analysis of combined short- and long-read data.

Sequence coverage is a major consideration during project planning, for both clinical and research projects. The DNAscope Hybrid pipeline can be used with a range of coverages, including targeted long-read sequencing, and is designed to fully utilize the available read data. In this paper, we benchmark full-coverage short-read datasets (at ∼35× coverage), with a range of long-read coverages to assess pipeline performance. Given the robust performance of the hybrid pipeline at a range of coverages, we believe that full-coverage short-read sequencing combined with either targeted or low-coverage (7× to 15×) long-read sequencing will be applicable to a wide range of projects, enabling high-accuracy SNVs and indels while also incorporating high-accuracy structural variants, which are not accessible from short-read data alone.

## Future directions

The DNAscope Hybrid pipeline is actively under development, with several planned improvements and expansions.

Somatic variant calling is an area where the hybrid method could provide significant advantages. Clinically relevant somatic variants often occur at low allele frequencies and may introduce homopolymer sequence repeats due to deficiencies in DNA error repair mechanisms. Somatic structural variants, including gene fusions, are important drivers of tumorigenesis. Accurately detecting these variants requires both high sequencing depth and long reads to resolve complex genomic regions. Currently, no single sequencing technology meets both requirements effectively. Hybrid approaches present a promising solution by combining the strengths of short- and long-read sequencing technologies, especially when combined with targeted sequencing of clinically relevant regions that are difficult to resolve from short-read data alone.

In addition to single-sample variant identification, DNAscope can process cohort samples through joint calling, utilizing pre-trained sequencing platform-specific models for short- or long-read data. This approach represents an alternative method of hybrid analysis across multiple sample types. In a recently published application note (Sentieon, 2025c), the DNAscope joint caller demonstrated its ability to harmonize different error patterns across multiple sequencing platforms, producing results that are fully interoperable with datasets generated from Illumina sequencers. Moving forward, we plan to collect additional short- and long-read datasets from the 1kGP (1000 Genome Project) and further benchmark the hybrid joint calling pipeline using these data.

Currently, this benchmark project includes datasets from only two sequencing platforms: Illumina and PacBio. However, several other commercially available platforms—particularly for short reads—are gaining traction. DNAscope already supports additional sequencing platforms, including Element Biosciences, Ultima Genomics, and Complete Genomics, among others. We anticipate that the hybrid pipeline will also be applicable to these and other emerging sequencing platforms. As part of our roadmap, we plan to develop and benchmark hybrid models for these additional sequencing platforms.

## Data Availability

All VCF files and CNV truth files have been uploaded to https://zenodo.org/records/15033928. FASTQ files were downloaded from publicly available sources: PacBio: Human whole-genome sequencing datasets from the Revio system for the Genome in a Bottle trio HG002 + HG003 + HG004, with one Revio SMRT Cell per sample replicate. Download link: https://downloads.pacbcloud.com/public/revio/2022Q4/. Illumina: pFDA Truth Challenge V, HG002 + HG003 + HG004. Download link: https://precision.fda.gov/challenges/10/intro. Benchmark small variant (SNV and indel) VCF files were downloaded from publicly available sources: GIAB v4.2.1 SNP/indel: https://ftp-trace.ncbi.nlm.nih.gov/ReferenceSamples/giab/release/AshkenazimTrio/. Draft Q100 v0.019: https://ftp-trace.ncbi.nlm.nih.gov/ReferenceSamples/giab/data/AshkenazimTrio/analysis/NIST_HG002_DraftBenchmark_defrabbV0.019-20241113/. CMRG v1.00: https://ftp-trace.ncbi.nlm.nih.gov/ReferenceSamples/giab/release/AshkenazimTrio/HG002_NA24385_son/CMRG_v1.00/GRCh38/SmallVariant/. Benchmark SV VCF files were downloaded from publicly available sources: Draft Q100 v0.019 SV: https://ftp-trace.ncbi.nlm.nih.gov/ReferenceSamples/giab/data/AshkenazimTrio/analysis/NIST_HG002_DraftBenchmark_defrabbV0.019-20241113/. CMRG v1.00: https://ftp-trace.ncbi.nlm.nih.gov/ReferenceSamples/giab/release/AshkenazimTrio/HG002_NA24385_son/CMRG_v1.00/GRCh38/StructuralVariant/.

## Abbreviations

AWS, Amazon Web Services; CLI, command line interface; CNV, copy-number variation; CMRG, challenging medically relevant genes; DV, DeepVariant; FP, false positive; FN, false negative; GBM, gradient boosting machine; GIAB, Genome in a Bottle; HLA, human leukocyte antigen; ILMN, Illumina sequencing data; NICU, neonatal intensive care unit; NGS, next-generation sequencing; ONT, Oxford Nanopore Technologies; PB, PacBio HiFi sequencing data; SV, structural variant (≥50 bp); T2T consortium, Telomere-to-Telomere consortium; TRHP, tandem repeat and homopolymer regions; WGS, whole-genome sequencing.
